# Spasticity, spastic dystonia, and static stretch reflex in hypertonic muscles of patients with multiple sclerosis

**DOI:** 10.1016/j.cnp.2021.05.002

**Published:** 2021-06-16

**Authors:** Luca Puce, Antonio Currà, Lucio Marinelli, Laura Mori, Elisabetta Capello, Rachele Di Giovanni, Matteo Bodrero, Claudio Solaro, Filippo Cotellessa, Francesco Fattapposta, Carlo Trompetto

**Affiliations:** a Department of Neuroscience, Rehabilitation, Ophthalmology, Genetics, Maternal and Child Health, University of Genova, Italy; b Academic Neurology Unit, A. Fiorini Hospital, Terracina (LT), Department of Medical-Surgical Sciences and Biotechnologies, Sapienza University of Rome, Polo Pontino, Italy; c Division of Clinical Neurophysiology, Department of Neuroscience, IRCCS Ospedale Policlinico San Martino, Genova, Italy; d Division of Neurorehabilitation, Department of Neuroscience, IRCCS Ospedale Policlinico San Martino, Genova, Italy; e Neurology Clinic, Department of Neuroscience, IRCCS Ospedale Policlinico San Martino, Genova, Italy; f Dept. of Rehabilitation, CRRF “Mons. Luigi Novarese”, Moncrivello, VC, Italy; g Neurology Unit, Policlinico Umberto I, Department of Neurology and Psichiatry, Sapienza University of Rome, Rome, Italy

**Keywords:** Electromyography (EMG), Stretch reflex, Muscle stretch, Muscle spindles, Spinal excitability, MS, multiple sclerosis, UMNS, upper motor neuron syndrome, DSR, dynamic stretch reflex, SSR, static stretch reflex, SD, spastic dystonia, MAS, modified Ashworth scale, MRC, medical research council scale, ARV, average rectified value

## Abstract

**Objective:**

To investigate prevalence of EMG patterns underlying hypertonia in multiple sclerosis (MS) and whether these patterns indicate different levels of spinal excitability.

**Methods:**

We investigated the EMG activity recorded from 108 hypertonic muscles of 59 consecutive MS patients. To investigate spastic dystonia (SD), we looked for the presence of EMG activity in muscles in a resting position. To investigate dynamic stretch reflex (DSR) and static stretch reflex (SSR), we looked for the presence of EMG activity in response to a manually performed passive stretch of the muscle.

**Results:**

DSR was evoked in 104 muscles. In 51 muscles, DSR was the sole EMG activity. This pattern corresponds to the classical notion of spasticity, and was predominant in extensors. In contrast, SSR was detected in 48 muscles – predominantly in flexors. SD was observed in 28 muscles, showing even distribution in flexor and extensor muscles. Only in the flexors, SSR was associated with a larger DSR compared to spasticity.

**Conclusions:**

These findings likely depend on the central effects of both flexor and extensor spindle afferents on the homonymous spinal motor neurons.

**Significance:**

Improving our capacity to assess spinal excitability in MS patients.

## 1 Introduction

In patients affected by chronic upper motor neuron syndrome (UMNS), one common clinical sign is muscle hypertonia, which is often velocity-dependent, i.e. greater resistance is experienced with fast stretches. Muscle hypertonia can hinder function, and result in pain and complications, limiting the potential benefit of rehabilitation.

In some cases, muscle hypertonia is caused by secondary soft tissue changes in muscles, tendons, and ligaments - a condition usually referred to as intrinsic hypertonia (Dietz et al., 1981). However, compelling evidence shows that in the overwhelming majority of UMNS patients, muscle hypertonia is due to increased stretch reflex activity (Thilmann et al., 1991) - a condition that can be called reflex hypertonia. Although only reflex hypertonia is reported to be velocity-dependent, intrinsic hypertonia and reflex hypertonia often co-exist within the same muscle (Sheean, 1998a), and it can be difficult to distinguish between the two forms in a clinical setting (Malhotra et al., 2008, O’Dwyer and Ada, 1996).

Stretch reflex is an involuntary muscle contraction in response to passive stretch. It can be studied using electromyography (EMG). When muscle length changes due to passive stretch, two phases can be distinguished: a dynamic phase, during which the muscle length varies, and a subsequent static phase, during which the muscle length remains constant while the muscle is stretched. Accordingly, the stretch reflex includes a dynamic stretch reflex (DSR), which is the muscle contraction produced by the dynamic phase of stretch, and a static stretch seflex (SSR), which is the muscle contraction produced by the static phase of stretch.

In fully relaxed healthy people, SSR cannot be evoked. DSR can only be evoked using rapid stretch velocities, for instance as produced by a sharp blow to the tendon. On the contrary, using a slow stretch velocity, as produced by the manual movements used to test muscle tone, DSR cannot be evoked (Burke, 1988, Sheean, 2002, Sheean, 1998b, Thilmann et al., 1991, Yeo et al., 1998). Therefore, in fully relaxed healthy people, muscle tone is considered to be determined exclusively by the mechanical properties of the limb (Burke, 1988, Rothwell, 1994).

Compared to healthy people, the situation is more complex in the hypertonic muscles of UMNS patients attempting to relax. Muscle stretch at rapid velocity evoked an exaggerated DSR, which may cause clonus. Additionally, in a large proportion of muscles, also the slower movement used to test muscle tone can elicit a DSR. Some of these muscles are completely relaxed prior to passive stretch and during the static phase of stretch, as shown by EMG studies. This condition is termed spasticity, and it is a dynamic phenomenon, i.e. present only during the dynamic phase of stretch (Burke, 1988, Burke, 1975, Hoefer and Putnam, 1940, Landau, 1969). This feature, together with the dependence on passive stretch velocity (i.e. greater EMG activity with faster stretches), is consistent with spasticity being mainly due to the discharge of Ia afferents from muscle spindles, which have a much higher firing rate during dynamic stretch than during static stretch (Matthews and Stein, 1969, Rothwell, 1994). However, in other cases, EMG of the hypertonic muscles of UMNS patients reveals both DSR and SSR (Sheean, 1998a, Trompetto et al., 2014).

In the hypertonic muscles of UMNS patients at rest, muscle responses to stretch are increased due to changes in the spinal excitability of the stretch reflex pathway. This has been demonstrated by microneurographic studies of spindle afferents, revealing no evidence of receptor hypersensitivity (Hagbarth et al., 1973, Macefield, 2013, Wilson et al., 1999). Among the spinal mechanisms that can generate stretch reflex hyperexcitability, some act on the presynaptic terminals of spindle afferents, while others act on spinal motor neuron membranes by reducing post-synaptic inhibition or by inducing denervation supersensitivity (for a review see Trompetto et al., 2014).

In addition to DSR and SSR, spontaneous EMG activity (i.e. not induced by stretch) is detected in some hypertonic muscles of UMNS patients. This condition has been termed spastic dystonia (SD), referring to the patients’ inability to voluntarily silence muscle activity on command (Trompetto et al., 2019b). This inability leads to spontaneous tonic muscle contractions that are stretch-sensitive and ultimately amplify the velocity-dependent hypertonia (Trompetto et al., 2014). SD is an efferent form of muscle overactivity that depends on continuous supraspinal drive to spinal motor neurons (Gracies, 2005); however, it may contribute to enhancing stretch reflex excitability by increasing the likelihood that spinal motor neurons will be excited by sensory inputs. Consequently, DSR evoked in SD-affected muscles exhibits a higher amplitude than that evoked in spasticity-affected muscles (Trompetto et al., 2019a). Spasticity and spastic dystonia are the two phenomena underlying reflex hypertonia. The basic feature that differentiates the two forms of reflex hypertonia is the sensory input role. In spasticity the sensory input acts as a trigger for α-motor neuron activation, while in spastic dystonia it acts as a modulator of α-motor neuron activity.

Neither SSR nor SD are triggered by the dynamic phase of stretch. Thus, they do not fulfil the above-given definition of spasticity. While there is an impressive volume of literature focused on spasticity, little attention has been paid to SD (Lorentzen et al., 2018, Marinelli et al., 2017) and even less to SSR in UMNS (Trompetto et al., 2014). While it is well known that several EMG patterns may be observed in the hypertonic muscles of UMNS patients (Sheean, 1998c, Trompetto et al., 2014), to our knowledge, no prior study has specifically examined their prevalence. Therefore, the prevalence rates of various EMG patterns of muscle hypertonia are unknown, as are the prevalence changes according to the involved muscle and UMNS aetiology. Furthermore, no data are available regarding whether, or the extent to which, the various EMG patterns may reflect different levels of spinal excitability.

In the present study, we investigated the EMG patterns underlying muscle hypertonia in a group of patients affected by multiple sclerosis (MS).

## 2 Materials and methods

### 2.1 Subjects

In this cross-sectional study, patients were consecutively enrolled at neuro-rehabilitation outpatient clinics according to the following inclusion criteria: MS diagnosed according to the revised McDonald’s criteria (Polman et al., 2011); age ≥ 18 years; sufficient cognitive functioning to give informed consent and to understand instructions (specifically, to remain relaxed during evaluation), as identified by a Mini-Mental Status Examination (MMSE) score of ≥ 24/30 (Folstein et al., 1975); and muscle hypertonia, indicated by a Modified Ashworth Scale (MAS) score of ≥ 1, affecting at least one of the following muscle groups: elbow, wrist, knee, and ankle flexors and extensors. The exclusion criteria were neurological conditions in addition to MS that may affect motor function, other medical conditions likely to interfere with the study protocol, use of intrathecal baclofen, and treatment with botulinum toxin within the last 8 months. Prior to patient enrolment, we obtained written informed consent, as approved by the local ethical committee (CER Liguria: 074REG2017).

### 2.2 Clinical assessment

We bilaterally rated the muscle tone of the elbow, wrist, knee, and ankle flexors and extensors according to the MAS (Bohannon and Smith, 1987). For hypertonic muscle groups (MAS ≥ 1), strength was rated according to the Medical Research Council (MRC) scale.

### 2.3 EMG and kinematic recordings

In hypertonic muscle groups (MAS ≥ 1), we investigated surface EMG (s-EMG) activity. If the tone of a muscle group was bilaterally increased, we investigated the more affected side. In accordance with SENIAM (Surface Electromyography for Non-Invasive Assessment of Muscles) guidelines (Hermens, 1999), a surface pre-amplified electrode with fixed inter-electrode distance (TSD150B, Biopac Systems Inc, USA) was placed over the muscle belly of the *biceps brachii* (elbow flexors), *triceps brachii* (elbow extensors), *flexor carpi radialis* (wrist flexors), *extensor carpi radialis* (wrist extensors), *vastus medialis* (knee extensors), *biceps femoris* (knee flexors), *tibialis anterior* (ankle flexors), and *soleus* (ankle extensors). Joint motion was recorded using a twin-axis electronic goniometer (TSD130B, Biopac Systems Inc, USA). All signals were acquired using an MP150 unit (Biopac Systems Inc, USA) with a 2-KHz sampling rate. A Blackman-61 dB 10–350 Hz band-pass filter was used for offline processing (AcqKnowledge 3.8.1 software; Biopac Systems Inc, USA).

### 2.4 Experimental protocol

Throughout the entire duration of the recording session, patients were instructed to stay completely relaxed and silent. The upper limbs were assessed with the patient lying supine on the bed. Lower limbs were assessed with the patient prone, keeping their feet off of the bed. SD was assessed first, immediately followed by evaluation of DSR and SSR.

#### 2.4.1 SD assessment

For SD assessment, the upper limbs were placed over the subject’s trunk, with the wrists in a neutral position and the elbows flexed to 90 degrees. For the lower limbs, to investigate EMG activity of ankle flexors and extensors, the feet were left in their natural position, which was halfway between maximum ankle flexion and maximum extension in the vast majority of patients. To investigate the knee flexors, the leg was kept passively flexed with the thigh at 90 degrees. These positions were chosen to keep the target muscle in an intermediate position between maximum flexion and maximum extension. The only exception was for analysis of knee extensors, where SD was assessed with the leg in full extension (natural position of the patient). The electrode and the goniometer were placed on the selected body segment, and then the EMG signal was recorded for 60 s.

#### 2.4.2 DSR assessment

After evaluating SD, the examiner grasped the selected body segment and moved it to the position of maximum flexion (to evaluate a flexor muscle) or to the position of maximum extension (to evaluate an extensor muscle). After a few seconds, the segment was again moved to the opposite position (from maximum flexion to maximum extension to evaluate flexors and *vice versa* to evaluate extensors) in 1 s (dynamic phase of the stretch). We applied a method developed in our laboratory to control the duration of passive displacement (Marinelli et al., 2013).

#### 2.4.3 SSR assessment

After the dynamic phase of the stretch, the patient’s segment was passively held in maximal flexion or maximal extension for 60 s (static phase of stretch).

### 2.5 Data analysis

We used the angle values detected by the electronic goniometer to calculate the onset and termination of the dynamic phase of the stretch. The times corresponding to stretch onset and termination were visually detected on the goniometer trace displayed on the computer screen, using a display gain of 20 degrees/cm and a temporal window of 340 ms/cm (Marinelli et al., 2017).

#### 2.5.1 Single-muscle analysis

For each muscle, we visually examined the unrectified EMG signal to look for the presence of SD, DSR, and SSR. SD and SSR were considered present if EMG activity was detected during most of the observation period (at least 40 s). DSR was considered present if movement-related EMG activity clearly stood out from the ongoing EMG recording during the dynamic phase of the stretch. We measured the DSR, SSR, and SD amplitudes by calculating the average rectified value (ARV, µV) of the corresponding EMG activity (Hermens, 1999). The ARVs of the SD and SSR were calculated from 6 consecutive bins of 10 s each.

#### 2.5.2 Analysis across muscles

For each tested muscle, EMG patterns were defined according to the presence of SD, DSR, and SSR. Differences between the clinical data (MAS and MRC scores) and ARVs in the different EMG patterns were analysed using the Mann-Whitney *U* test. To investigate the time-courses of SD-ARV and SSR-ARV, we used repeated-measures ANOVA with bins as the within-subjects factor. Bonferroni *post-hoc* comparison was performed when appropriate. Differences were considered significant when *P* < 0.05. All measures of variability are reported as standard deviation.

## 3 Results

The study enrolled 59 MS patients (30 women; mean age, 51 ± 10 years; range, 27–76 years). Table 1 shows the patients’ demographic and clinical features. An MAS score of ≥ 1 was found in ankle extensors in 50 patients (85%), knee extensors in 26 patients (44%), knee flexors in 19 patients (32%), wrist flexors in 6 patients (10%), elbow flexors in 5 patients (8%), and elbow extensors in 2 patients (3%). No patient had an MAS score of ≥ 1 in ankle flexors or wrist extensors. Overall, we identified a total of 108 hypertonic muscles, which were investigated using s-EMG.

**Table 1:** Patients’ demographic and clinical features.

Patient number	Age	Sex	Disease course	EDSS	Muscle strength (MRC score)						Muscle tone (MAS score)						Drugs for spasticity
					AE	KE	KF	WF	EF	EE	AE	KE	KF	WF	EF	EE	
1	59	M	PP	8	0	5		2	4		4	1		1	2		Gabapentin, Baclofen
2	56	M	PP	6	5						2						Nabiximols
3	34	M	SP	2	5						2						
4	54	F	SP	7	2	5					2	2					Gabapentin
5	53	M	PP	6	5	5					3	1					
6	46	M	PP	8	1	0	1		5		4	4	2		1		Nabiximols, Baclofen
7	35	F	SP	6	5						3						Tizanidine, Gabapentin
8	50	M	SP	6	5	5					3	3					
9	47	M	PP	4	5						2						
10	42	F	SP	6.5	3	3			4	4	2	3			2	1	Cannabis
11	52	M	SP	6	5						1						
12	60	M	SP	6	5						4						
13	47	F	SP	7	5	5					4	1					
14	52	F	SP	6	0						4						
15	49	M	PP	7	3						4						Baclofen
16	41	F	RR	5.5	2	5					2	2					Baclofen, Nabiximols
17	51	M	SP	6.5	5						4						Baclofen
18	57	M	SP	7	5						4						
19	73	F	SP	8	3		4				4		4				
20	44	F	PP	5	5	5					3	3					Nabiximols, Tizanidine
21	55	F	SP	7.5	NR	0	0				4	3	3				Baclofen
22	51	F	PP	6.5	4						3						
23	65	F	PP	6.5	5						4						
24	52	F	PP	6.5	5			5			2			1			Gabapentin
25	53	F	PP	6	5	5					3	1					Gabapentin
26	33	F	PP	5	5						2						Nabiximols
27	71	F	SP	7.5		5						3					
28	76	M	SP	7	NR	1	1	4	1		4	1	1.5	3	3		
29	52	F	SP	6.5		5						3					Gabapentin
30	53	F	PP	7		2	0					3	2				
31	47	M	PP	8	2	4	0				3	2	1				
32	65	M	SP	8	0	5					2	3					
33	51	F	RR	4			5						1				
34	32	F	SP	4.5	5	5					2	1					Baclofen
35	35	F	SP	6	5						3						Gabapentin
36	54	F	RR	6	5						1						
37	48	M	PP	8							1						Cannabis
38	48	M	PP	7			2						1				
39	53	M	SP	8				0	1					2	3		
40	56	M	SP	7.5		3						2					
41	56	F	RR	5	3	5	5	5				1	1.5	1			
42	54	F	SP	6	4	4	4				1	2	2				
43	44	M	RR	3	5						1.5						Baclofen
44	58	M	SP	6.5	4		5				1		1.5				
45	31	M	RR	6.5	3	3	3				4	1	4				
46	55	F	SP	7	4		3				2		1.5				
47	64	M	PP	5	5		4				2		2				Nabiximols
48	64	F	RR	2	NR						1						
49	31	F	RR	2	4						1						
50	55	F	SP	6.5	4						1						Nabiximols
51	54	M	RR	1.5	5		5				1		1				
52	63	M	PP	6		5	5					2	2				Baclofen, Pregabalin
53	57	M	RR	6	4	5	4				2	3	2				
54	52	M	SP	6	4		5				2		1				
55	42	F	SP	4	5						2						
56	27	M	SP	7.5	2	2	2				4	4	3				Cannabis
57	54	F	RR	5	3	5		4		4	3	1		3		1	
58	55	M	RR	4	5						1.5						
59	53	F	PP	6.5	4						2						Gabapentin

AE = ankle extensors; KE = knee extensors: KF = knee flexors; WF = wrist flexors; EF = elbow flexors; EE = elbow extensors; NR = not reported; SP = secondary progressive; PP = primary progressive; RR = relapsing-remitting; MAS = Modiﬁed Ashworth Scale; MRC = Medical Research Council Scale for strength; EDSS = Expanded Disability Status Scale.

DSR was evoked in 96% of muscles (*n* = 104). In the four muscles lacking DSR, no other EMG activity was detected. SSR was evoked in 44% of muscles (*n* = 48), and SD in 26% (*n* = 28). In all subjects with SD and/or SSR, the corresponding EMG activity was tonic and lasted for the entire duration of recording time.

### 3.1 EMG patterns in hypertonic muscles: DSR-alone, DSR + SSR, SD + DSR + SSR, and SD + DSR (Table 2)

DSR-alone was found in 47% of muscles (*n* = 51). This was the predominant pattern in the *soleus* muscle (68% patients) and in the *rectus femoris* muscle (50% patients), and was evoked in 1 of the 2 *triceps brachii* muscles examined. On the other hand, the DSR-alone pattern was evoked in the *biceps femoris* in only 16% of patients, and was absent from all examined *flexor carpi radialis* (*n* = 6) and *biceps brachii* (*n* = 5) muscles.

As part of two distinct EMG patterns (DSR + SSR and SD + DSR + SSR), we detected SSR in 44% of muscles (*n* = 48). SSR was predominant in the *biceps femoris* (79% patients), *flexor carpi radialis* (83% patients), and *biceps brachii* (80% patients) muscles. In contrast, SSR was found in the *soleus* muscle in only 30% of patients, and in the *rectus femoris* muscle in 35% of patients. Of the two patients with hypertonic elbow extensors, neither exhibited SSR in the *triceps brachii* muscle.

As part of two distinct EMG patterns (SD + DSR + SSR and SD + DSR), SD was found in 26% of muscles (*n* = 28). In the lower limbs, SD was detected in the *soleus* in 24% of patients, in the *rectus femoris* in 19% of patients, and in the *biceps femoris* in 26% of patients. In the upper limbs, SD was found in the *flexor carpi radialis* in 50% of patients, and in the *biceps brachii* muscle in 40% of patients. Of the two patients with hypertonic elbow extensors, only one exhibited SD in the *triceps brachii* muscle. All 10 flexor muscles exhibiting SD, also showed SSR. In contrast, of the 18 extensor muscles showing SD, only 13 (72%) also showed SSR. SD but not SSR (the SD + DSR pattern) was observed in four *rectus femoris* and one *triceps brachii* muscles.

**Fig. 1:**
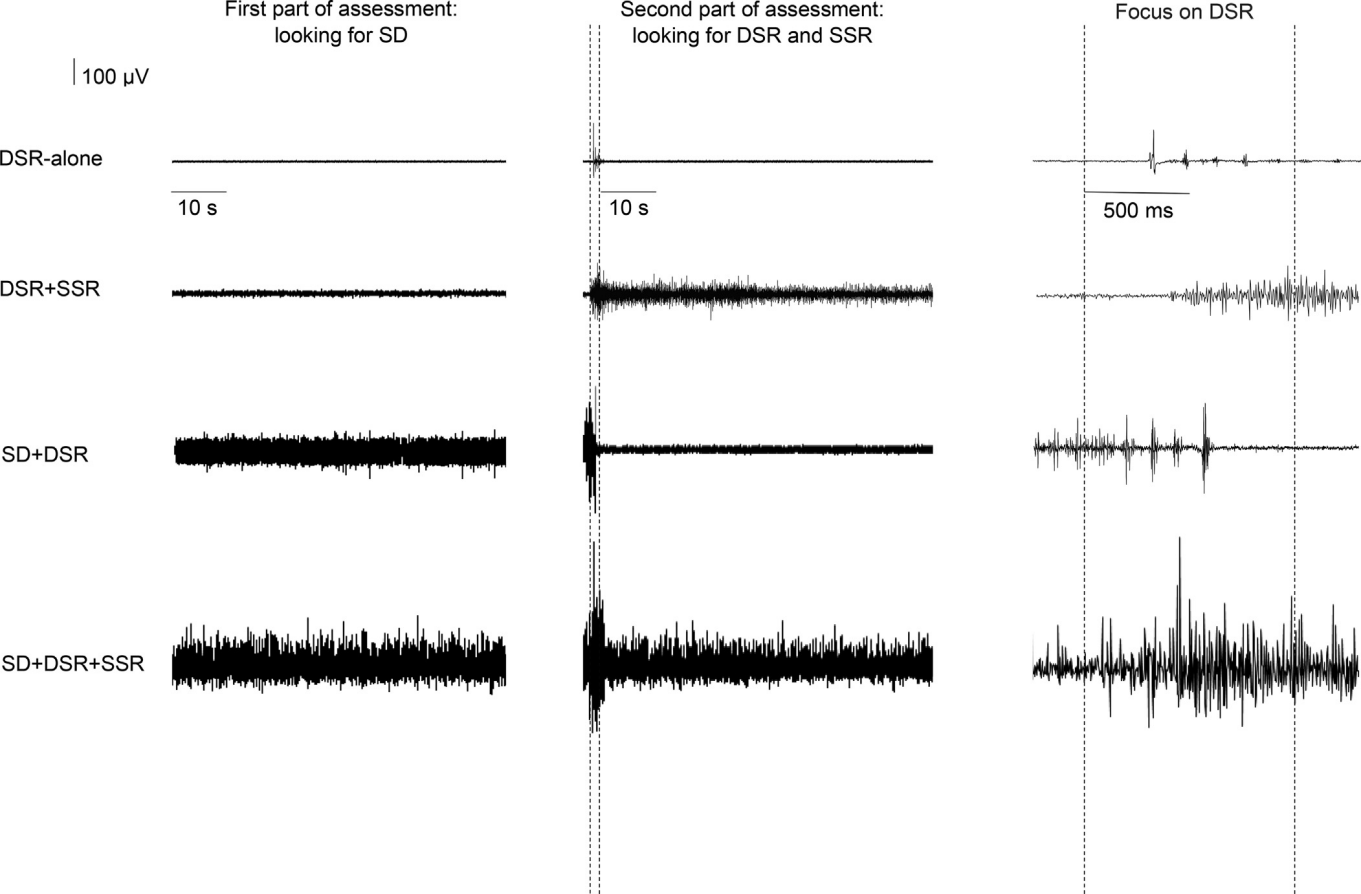
Raw data recorded from representative patients displaying the various EMG patterns recorded in the rectus femoris muscle. DSR = dynamic stretch reflex; SD = spastic dystonia; SSR = static stretch reflex. The first column shows raw data recorded during SD assessment. The second column presents raw data recorded during DSR and SSR assessment. The vertical dotted lines define the dynamic phase of the stretch (from maximal extension to maximal flexion of the leg) (time interval, 1 s). Raw data recordings of static phase of stretch (rectus femoris muscle kept in an elongated position) are shown to the right of the second vertical dotted line. The third column shows an enlargement of the DSR raw data displayed in the second column.

**Fig. 2:**
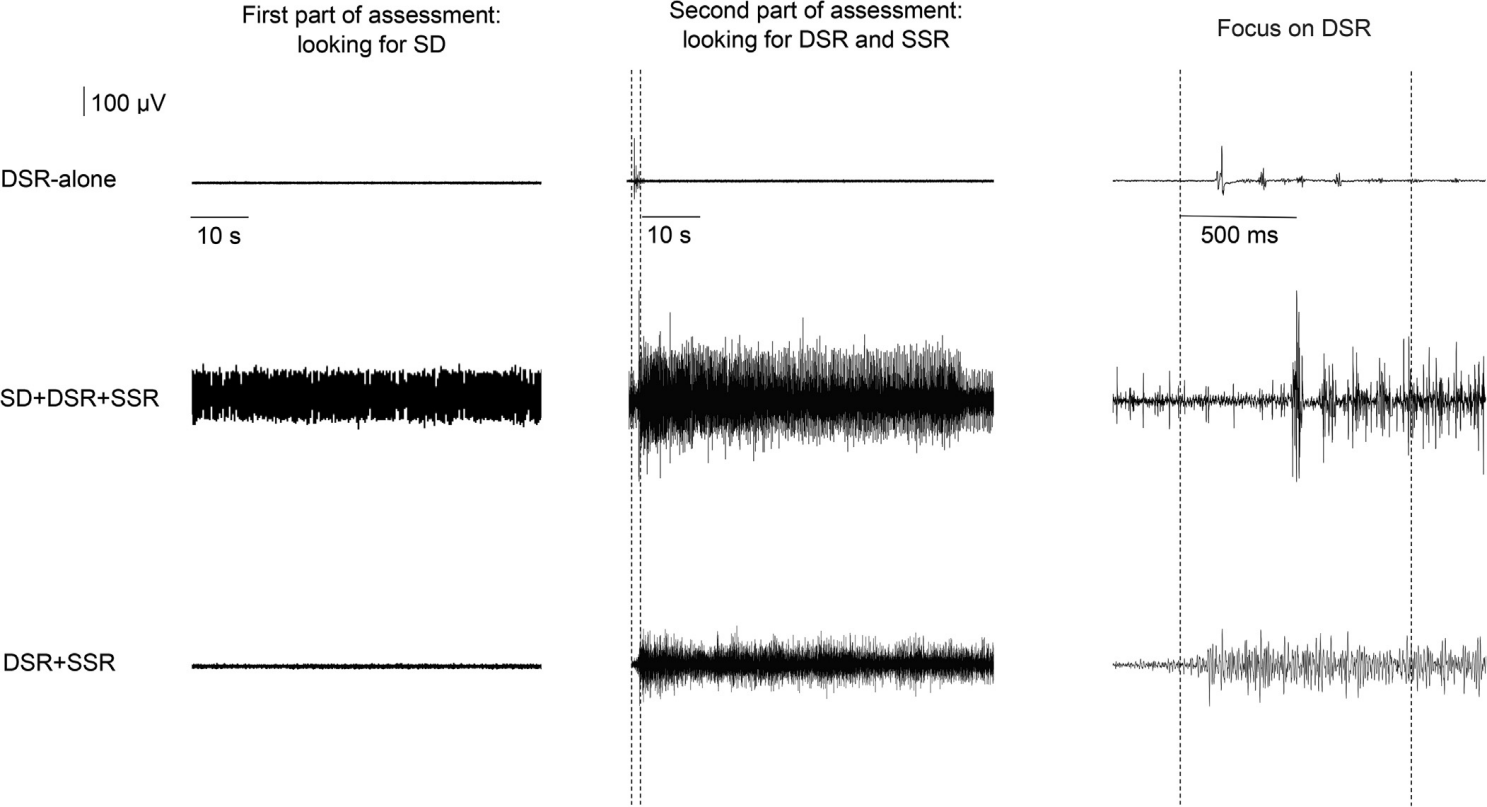
Raw data recorded from representative patients displaying the various EMG patterns recorded in the biceps femoris muscle. DSR = dynamic stretch reflex; SD = spastic dystonia; SSR = static stretch reflex. The first column shows raw data recorded during SD assessment. The second column presents raw data recorded during DSR and SSR assessment. The vertical dotted lines define the dynamic phase of the stretch (from maximal flexion to maximal extension of the leg) (time interval, 1 s). Raw data recordings of static phase of stretch (biceps femoris muscle kept in an elongated position) are shown to the right of the second vertical dotted line. The third column shows an enlargement of the DSR raw data displayed in the second column.

Table 2 shows the EMG patterns in all examined hypertonic muscles. Fig. 1 and Fig. 2 show raw data from representative patients, indicating the various EMG patterns recorded in the *rectus femoris* and *biceps femoris* muscles.

**Table 2:** EMG patterns in each one of the six muscle groups in which hypertonia was detected.

				EMG patterns			
Muscle							
		DSR-alone	DSR+SSR	SD+DSR+SSR	SD+DSR	No EMG	Total number
Soleus	Subjects’ number (%) ARV SD ARV DSR ARV SSR	34 (68%) 12.7 ± 9.5	3 (6%) 16.0 ± 8.2 8.9 ± 6.6	12 (24%) 12.1 ± 5.4 35.0 ± 14.1 27.3 ± 13.8	0	1 (2%)	50
Rectus Femoris	Subjects’ number (%) ARV SD ARV DSR ARV SSR	13 (50%) 16.2 ± 7.3	8 (31%) 15.3 ± 6.4 8.4 ± 4.8	1 (4%) 32.8 41.0 18.2	4 (15%) 18.8 ± 6.6 38.5 ± 9.3	0	26
Triceps brachii	Subjects’ number (%) ARV SD ARV DSR ARV SSR	1 (50%) 14.9	0	0	1 (50%) 4.8 5.7	0	2
Biceps Femoris	Subjects’ number (%) ARV SD ARV DSR ARV SSR	3 (16%) 12.1 ± 5.8	10 (53%) 24.9 ± 9.0 20.3 ± 10.8	5 (26%) 14.4 ± 3.7 28.2 ± 6.9 25.6 ± 15.1	0	1 (5%)	19
Flexor carpi radialis	Subjects’ number (%) ARV SD ARV DSR ARV SSR	0	2 (33%) 16.0 ± 7.0 11.7 ± 4.0	3 (50%) 8.5 ± 4.7 13.7 ± 8.4 10.8 ± 5.6	0	1 (17%)	6
Biceps brachii	Subjects’ number (%) ARV SD ARV DSR ARV SSR	0	2 (40%) 17.5 ± 7.8 22.8 ± 18.5	2 (40%) 7.7 ± 2.0 15.5 ± 3.5 14.2 ± 11.1	0	1 (20%)	5
Total number		51	25	23	5	4	108

Means and standard deviation of the Average Rectified Value (ARV) of spastic dystonia (SD), dynamic stretch reflex (DSR) and static stretch reflex (SSR).

### 3.2 EMG patterns and DSR amplitude in each muscle group

Examinations of the *soleus* muscle revealed 12 patients with SD, in whom the DSR amplitude (35.0 ± 14.1 µV) was higher than that in the 34 patients with DSR-alone (12.7 ± 9.5 µV; *P* < 0.0001) and in the three patients with DSR + SSR (16.0 ± 8.2 µV; *P* = 0.04). DSR amplitude did not differ between DSR-alone and DSR + SSR (*P* = 0.3).

With regards to the *rectus femoris* muscle, five patients exhibited SD. The DSR amplitude in these patients (39.0 ± 8.1 µV) was higher than that recorded in the 13 patients with DSR-alone (16.2 ± 7.3 µV; *P* = 0.002), and in the eight patients with DSR + SSR (15.3 ± 6.4 µV; *P* = 0.005). DSR amplitude did not differ between DSR-alone and DSR + SSR (*P* = 0.7).

*Biceps femoris* muscle examinations showed that the DSR amplitude in the five patients with SD (28.2 ± 6.9 µV) was higher than that in the three patients with DSR-alone (12.1 ± 5.8 µV; *P* = 0.03), but did not differ from that in the 10 patients with DSR + SSR (24.9 ± 9.0 µV; *P* = 0.5). Furthermore, DSR amplitude was higher in the patients with DSR + SSR than in the three patients with DSR-alone (*P* = 0.04).

Analysis of the *biceps brachii* muscle revealed DSR amplitudes of 15.5 ± 3.5 µV in the two patients with SD, and 17.5 ± 7.8 µV in the two patients with DSR + SSR. *Flexor carpi radialis* muscle examinations showed DSR amplitudes of 13.7 ± 8.4 µV in the three patients with SD, and 16.0 ± 7.0 µV in the two patients with DSR + SSR. Finally, examination of the *triceps brachii* muscle revealed DSR amplitudes of 5.7 µV in the patient with SD, and 14.9 µV in the patient with DSR-alone.

### 3.3 Comparison of clinical data (MAS and MRC scores) among EMG patterns

MAS scores did not differ between patients showing distinct EMG patterns. MRC scores were higher in patients with DSR-alone (4.0 ± 1.4) than in patients with SD + DSR + SSR (3.0 ± 1.8; *P* = 0.02). All other comparisons yielded non-significant findings.

### 3.4 Time-course of SD and SSR

**Fig. 3:**
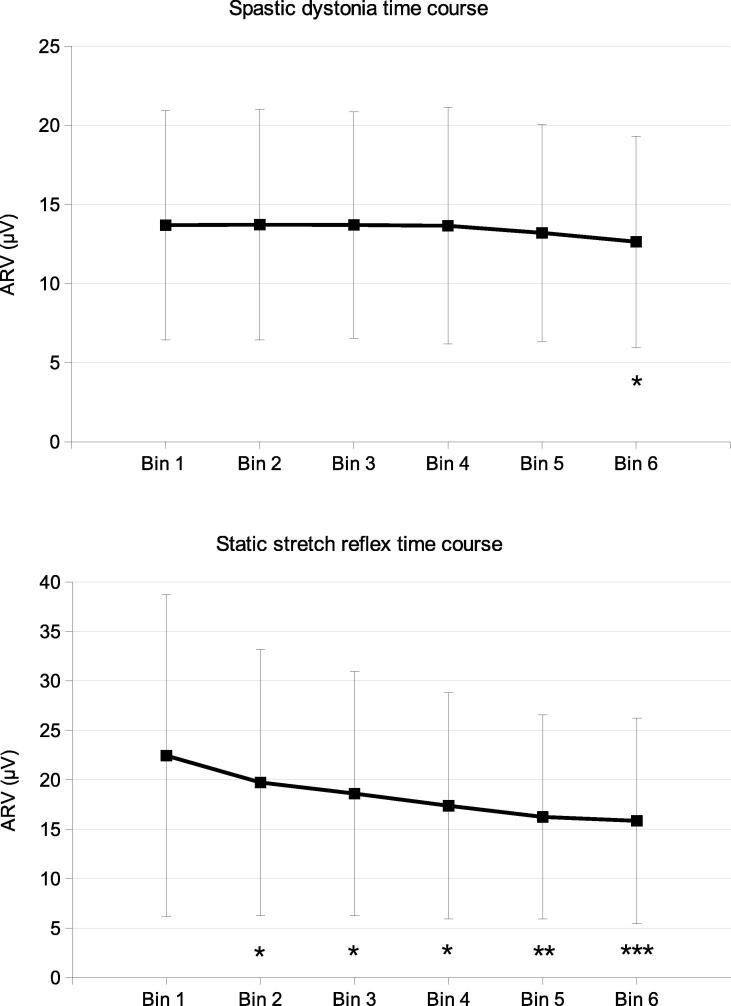
Mean ± standard deviation of the average rectified values of SD and SSR. (A) Data from the 24 muscles in which SD was investigated for 60 s. Bin 1: 0–10 s; bin 2: 10–20 s; bin 3: 20–30 s; bin 4: 30–40 s; bin 5: 40–50 s; bin 6: 50–60 s. * P < 0.05 compared to bins 1–2-3–4. (B) Data from the 42 muscles in which SSR was investigated for 60 s. Bin 1: 0–10 s; bin 2: 10–20 s; bin 3: 20–30 s; bin 4: 30–40 s; bin 5: 40–50 s; bin 6: 50–60 s. *** P < 0.05 compared to bins 1–2-3; ** P < 0.05 compared to bins 1–2; * P < 0.05 compared to bin 1.

We analysed all muscles with SD recorded on EMG for at least 1 min (*n* = 24, 4 *soleus* muscles were excluded because SD was erroneously recorded for < 1 min). The results showed that SD amplitude decreased across bins (F[5,115] = 5.9; *p* < 0.0001). We also analysed all muscles with SSR recorded on EMG for at least 1 min (*n* = 42, 4 *soleus* and 2 *rectus femoris* muscles excluded because SSR was erroneously recorded for < 1 min), and found that SSR amplitude decreased across bins (F[5,205] = 17.7; *p* < 0.0001) (Fig. 3).

## 4 Discussion

### 4.1 Clinical findings

We examined 108 hypertonic muscles (MAS score ≥ 1), of which 95 were lower limb muscles, confirming the greater prevalence of hypertonia in the lower limbs among MS patients (Flachenecker et al., 2014). This hypertonia distribution likely reflects the predominant lesion dissemination. MS involves all levels of the central nervous system, and the probability of involvement of a functional pathway increases with increasing pathway length.

Among the 95 hypertonic muscles of the lower limbs, 76 were extensors and 19 flexors. In contrast, among hypertonic muscles of the upper limbs, 11 were flexors and only two were extensors. The predominance of hypertonia in the upper limb flexors and lower limb extensors confirms the general assumption that the muscles most commonly affected in UMNS patients are those resisting gravity, as dictated by human standing posture.

In healthy subjects, counteracting the force of gravity leads anti-gravity muscles to be larger, stronger and more reflexively excitable than their antagonists. In clinical neurology, deep tendon reflexes are more easily evoked in anti-gravity muscles; in clinical neurophysiology the H reflex - a way to assess the excitability of the stretch reflex (Schieppati, 1987) – can be elicited at rest only in anti-gravity muscles (Burke, 2016). In patients affected by UMNS these peculiarities are maintained, and anti-gravity muscles are less weak than their antagonists while exhibiting greater excitability of the stretch reflex (and, consequently greater reflex hypertonia).

### 4.2 EMG findings

Of the 108 muscles examined, four exhibited no EMG activity. These muscles were likely affected exclusively by intrinsic hypertonia, without a neural component (Dietz et al., 1981). However, it cannot be excluded that these may have been normal muscles with overestimated tone scores, since the MAS score was 1 or 2 in all four muscles.

Each of the remaining 104 muscles exhibited a DSR, confirming the existence of a reflex component in the vast majority of hypertonic muscles in UMNS patients (Thilmann et al., 1991). DSR-alone, i.e. spasticity, was the predominant EMG pattern in extensor muscles, while SSR was prevalent in flexor muscles. Distinctly, SD was evenly distributed in flexor and extensor muscles. SD may have been more prevalent in the upper limbs; however, these data must be cautiously interpreted, given the small sample size of upper limb muscles.

In our patients with MS, SSR decreased over time, confirming our recent findings in the hamstrings (Trompetto et al., 2020). The decrease of this EMG activity, as the decrease of SD found in the present study, most likely parallels the decrease in spinal excitability, which occurs when the subject is relaxed and without disturbing external stimuli. Unfortunately, our single s-EMG recordings cannot exclude a concomitant decrease of the spindles discharge over time at static muscle lengths. In stroke patients, we found a similar decrease of SSR over time (Trompetto et al., 2019a), while Forman et al (2019) found a decrease in patients with cerebral palsy, but not in stroke patients. We agree with the Authors that, in the two studies, the different speed of passive muscle lengthening (before the static phase) can explain this discrepancy (Forman et al., 2019). We also agree with these Authors that the role of reflex afferent circuits in sustained muscle activity during the static phase of stretch needs further investigations (Forman et al., 2019, Trompetto et al., 2014).

As expected, and confirming our prior observations in stroke patients (Trompetto et al., 2019a), the DSR size was always greater in muscles with SD than in muscles with spasticity. This is because spastic muscles are by definition relaxed prior to stretch, whereas muscles with SD are not. Indeed, SD activates the muscles tonically, despite all subjects’ attempts to wilfully terminate this involuntary activation. Therefore, spinal motor neurons targeting tonically activated muscles are excited more intensely and more easily by sensory inputs. Furthermore, although we acknowledge that activation of group I and II afferents may be non-linear, and that the α-γ linkage is largely hypothetical, this model predicts that the discharge of spindle afferents from contracting muscles is larger than that from relaxed muscles (Rothwell, 1994).

Because hypertonia is at least partly due to the DSR, muscles with SD were expected to be more hypertonic than those with spasticity. However, this was not the case due to their similar MAS scores. In contrast, muscle weakness (as measured by MRC score) was worse in muscles with SD than in those with spasticity. This is an interesting finding because, on one hand, it suggests that these two clinical signs may share some neural mechanisms and, on the other hand, it confirms that EMG assessment exhibits greater sensitivity than clinical scales for quantifying muscle overactivity in chronic UMNS subjects *(*Kumar et al., 2006, Malhotra et al., 2008*)*.

In flexor muscles, the EMG pattern DSR + SSR was characterised by a DSR larger than that found in spasticity, and similar to that found in SD. In contrast, in extensor muscles, the DSR + SSR pattern was characterised by a DSR of similar size to that found in spasticity, and smaller than that found in SD. SSR always accompanied SD in flexors, but this was not the case in extensors. Divergent combinations of the type of muscle action (flexor vs. extensor), DSR size (small vs. large), and presence vs. absence of SSR and SD would reasonably depend on the modality of spindle ending activation during the dynamic and static phases of muscle stretch, as well as the specificity of the effects that spindle endings exert on homonymous spinal motor neurons. During the dynamic phase of stretch, primary endings fire at a much higher rate than secondary endings, and this difference increases with the stretch velocity. When the dynamic phase ends, the discharge of primary endings declines, while the discharge of secondary endings exhibits little or no change. Consequently, when the muscle is maintained lengthened in the static phase of stretch, both types of spindle endings exhibit approximately the same firing rate (Brown et al., 1965, Rothwell, 1994). Both primary and secondary endings from flexor muscles facilitate homonymous motor neurons (i.e. those innervating the muscle from which the endings themselves originate). Differently, primary endings from extensor muscles facilitate homonymous motor neurons, while secondary endings from extensor muscles inhibit homonymous motor neurons (Burke et al., 1971, Burke et al., 1970, Hunt, 1952).

In flexor muscles, SSR was highly prevalent. In these muscles both primary and secondary spindle endings act synergistically onto homonymous motor neurons to produce muscle activation. Indeed, despite the reduced discharge of primary endings at the end the dynamic phase, the discharge of synergic secondary endings proved sufficient to maintain spinal motor neuron activation. In the minority of muscles exhibiting spasticity, only the dynamic phase of stretch (i.e. movement) produced reflex activity. In this situation, where more intense afferent input is needed to activate the motor neurons, spinal excitability must be lower than in muscles with SSR. This was reflected by the finding that the DSR size was smaller in flexors with spasticity (i.e. DSR-alone) than that in flexors with the SD + DSR + SSR or DSR + SSR pattern.

In extensor muscles, spasticity was the predominant pattern. In these muscles primary and secondary endings act as antagonists towards homonymous motor neurons. It is reasonable to hypothesize that after the dynamic phase, the reduced discharge of primary endings leaves the inhibitory effect of secondary endings unopposed, thereby preventing SSR in most of the examined muscles, and thus configuring spasticity. If this is true, the emergence of SSR in an extensor muscle would merely reflect the predominant facilitation of motor neurons induced by unbalanced actions of the primary endings. Our data fit this hypothesis well, and show that SSR may be absent in extensor muscles even in the presence of SD, a condition that increases the excitability of the stretch reflex circuitry (Marinelli et al., 2017, Trompetto et al., 2019a). Furthermore, in extensor muscles showing the DSR + SSR EMG pattern, the DSR size was similar to that found in spasticity, and smaller than that found in SD.

Overall, our data suggest that SSR in flexor muscles reflects higher spinal excitability of the stretch reflex circuitry than that acting in spasticity. On the other hand, the presence of SSR in extensor muscles reflects an imbalance that favours facilitation exerted by primary endings over inhibition exerted by secondary endings.

This study has potential limitations. First, the EMG patterns defined at rest give no insight into whether the phenomena contribute to the patient's disability. These results are preparatory to the study of the relationship between the EMG patterns underlying hypertonia and the degree of disability determined by them. In fact, to know if it is worth identifying these EMG patterns in MS patients with hypertonia, we will need to understand if they are associated with different levels of disability. Second, many of the patients examined were being treated with symptomatic oral drugs for spasticity. Although we acknowledge they may have changed the excitability of the stretch reflex, we consider it unlikely that the drugs exerted different effects on flexors and extensors, so as to impact the present results. Third, patients were not stratified by MS severity. To carry out this analysis, it will be necessary to design a study with a much larger sample than the 59 patients enrolled here.

### 4.3 Conclusions

Our present results showed that in hypertonic patients with MS, spasticity (DSR-alone) predominated in extensors, and was rare in flexors. This finding was paralleled by a higher frequency of SSR in flexors than in extensors. SSR in flexors reflects increased spinal excitability of the stretch reflex circuitry, but this is not the case with SSR in extensors. These findings likely depend on the distinct reflex actions that flexor and extensor secondary endings exert on the homonymous spinal motor neurons. In contrast, SD reflects increased stretch reflex excitability in both flexor and extensor muscles, between which SD was evenly distributed. These findings will help predict the level of spinal excitability based on the EMG pattern recorded from hypertonic muscles, depending on the muscle action, DSR size, and presence of SSR and SD. Our present observations open a path towards evaluating how time and/or treatment may influence spinal excitability in UMNS patients.

## Declaration of Competing Interest

The authors declare that they have no known competing financial interests or personal relationships that could have appeared to influence the work reported in this paper.
